# Phosphatidylserine is a global immunosuppressive signal in efferocytosis, infectious disease, and cancer

**DOI:** 10.1038/cdd.2016.11

**Published:** 2016-02-26

**Authors:** R B Birge, S Boeltz, S Kumar, J Carlson, J Wanderley, D Calianese, M Barcinski, R A Brekken, X Huang, J T Hutchins, B Freimark, C Empig, J Mercer, A J Schroit, G Schett, M Herrmann

**Affiliations:** 1 Department of Microbiology, Biochemistry and Molecular Genetics, Cancer Center, Rutgers New Jersey Medical School, 205 South Orange Ave, Newark, NJ 07103, USA; 2 Department of Internal Medicine 3—Rheumatology and Immunology, Friedrich-Alexander-Universität Erlangen-Nürnberg (FAU), University Hospital Erlangen, 91054 Erlangen, Germany; 3 Peregrine Pharmaceuticals, 14282 Franklin Avenue, Tustin, CA 92780, USA; 4 Universidade Federal do Rio de Janeiro, Rio de Janeiro, Brazil; 5 Laboratório de Biologia Celular, Instituto Oswaldo Cruz, Rio de Janeiro, Brazil; 6 Division of Surgical Oncology, Department of Surgery, Hamon Center for Therapeutic Oncology Research, Dallas, TX 75390-8593, USA; 7 Department of Pharmacology, University of Texas Southwestern Medical Center, Dallas, TX 75390-8593, USA; 8 Medical Research Council Laboratory for Molecular Cell Biology, University College London, Gower Street, London WC1E 6BT, UK; 9 Simmons Cancer Center and the Department of Immunology, University of Texas Southwestern Medical Center, Dallas, TX 75390 USA

## Abstract

Apoptosis is an evolutionarily conserved and tightly regulated cell death modality. It serves important roles in physiology by sculpting complex tissues during embryogenesis and by removing effete cells that have reached advanced age or whose genomes have been irreparably damaged. Apoptosis culminates in the rapid and decisive removal of cell corpses by efferocytosis, a term used to distinguish the engulfment of apoptotic cells from other phagocytic processes. Over the past decades, the molecular and cell biological events associated with efferocytosis have been rigorously studied, and many eat-me signals and receptors have been identified. The externalization of phosphatidylserine (PS) is arguably the most emblematic eat-me signal that is in turn bound by a large number of serum proteins and opsonins that facilitate efferocytosis. Under physiological conditions, externalized PS functions as a dominant and evolutionarily conserved immunosuppressive signal that promotes tolerance and prevents local and systemic immune activation. Pathologically, the innate immunosuppressive effect of externalized PS has been hijacked by numerous viruses, microorganisms, and parasites to facilitate infection, and in many cases, establish infection latency. PS is also profoundly dysregulated in the tumor microenvironment and antagonizes the development of tumor immunity. In this review, we discuss the biology of PS with respect to its role as a global immunosuppressive signal and how PS is exploited to drive diverse pathological processes such as infection and cancer. Finally, we outline the rationale that agents targeting PS could have significant value in cancer and infectious disease therapeutics.

## Facts

- PS externalization during apoptosis and cell stress are mediated by scramblases Xkr8 and TMEM16, respectively.
- Exposed PS is an evolutionarily conserved anti-inflammatory and immunosuppressive signal.
- An astonishing number of pathogens causing major infectious diseases utilize PS and apoptotic mimicry to evade host immune responses.
- PS signaling is highly dysregulated in the tumor microenvironment and autoimmune diseases.
- PS-targeting therapeutics (e.g., AnxA5, bavituximab) can stimulate immune activity.

## Open Questions

- Is PS dysregulation a universal mechanism of immune evasion for bacteria, viruses and protists?
- Should PS targeting be considered a global therapeutic option for infectious diseases?
- Should PS be considered a global checkpoint inhibitor for cancer?
- Are all PS signaling equally immunosuppressive?
- Are cofactors involved?

Many critical biochemical pathways require the presence of specific phospholipid classes in the inner and outer leaflet of the plasma membrane. Virtually all eukaryotic cells have an asymmetric distribution of phospholipids across their bilayer membrane, where the choline-containing phospholipids, phosphatidylcholine (PC) and sphingomyelin are predominately maintained on the outer membrane leaflet, and the amino-phospholipids (phosphatidylserine (PS) and phosphatidylethanolamine (PE)) are predominately localized in the inner membrane leaflet.^1^ This asymmetry is actively maintained by the regulated activity of ATP-dependent lipid transporters. However, membrane asymmetry collapses under a variety of physiological and pathological conditions resulting in dramatic changes in the biochemical properties of the membrane. For example, the redistribution of PS to the external face of the plasma membrane flags cells for their recognition, phagocytosis,^2^ and ultimate degradation by phagocytes (efferocytosis). Moreover, the interaction between PS-expressing cells and immune cells elicits profound immunological consequences by triggering immunosuppressive pathways that prevent both local and systemic immune activation. Although these pathways are used by apoptotic cells to quell potential immune sequelae against ‘self', these same pathways are hijacked by pathogens and tumors to promote their sinister life-threatening expansion. Taken together, these observations suggest that PS functions as an upstream immune checkpoint that suppresses the development of immunity. This raises the possibility that PS blockade by the therapeutic administration of PS-targeting agents can restore pathogen and tumor immunity.

## PS Asymmetry in Biological Membranes

**Figure 1:**
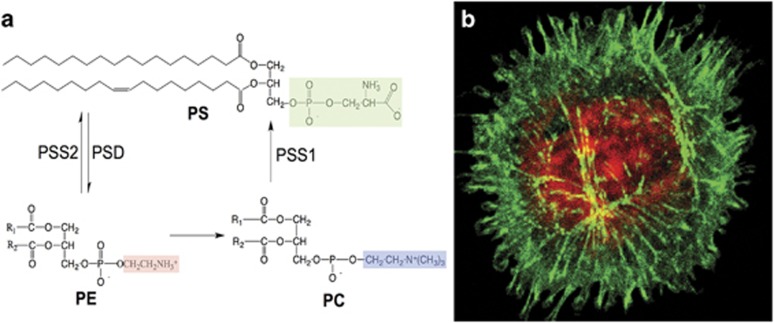
Molecular structure of PS and major biosynthetic and degradative pathways: (**a**) PS is comprised of a glycerol backbone esterified at the *sn*-1 and *sn-*2 carbons of the glycerol moiety with two fatty acyl chains of variable length and saturation, as well as a phosphate linkage at the *sn*-3 position covalently linked to serine (**a**). In eukaryotic cells, PS is synthesized from phosphatidylcholine (PC) and phosphatidylethanolamine (PE) by PSS1 and PSS2, respectively, and can be catabolized by phosphatidylserine decarboxylase (PSD) to generate PE. (**b**) During apoptosis and cell stress, PS is externalized to the outer surface of the plasma membrane, where it can be detected by fluorophores such as FITC-annexin V or GFP-lactadherin 3 (green). Red staining indicates Rhod-2AM that monitors intracellular Ca2+ levels, which are elevated during apoptosis and during cell stress

PS, the most abundant negatively charged glycerophospholipid in eukaryotic membranes, is comprised of a glycerol backbone esterified at the *sn*-1 and *sn-*2 carbons of the glycerol moiety with two fatty acyl chains of variable length and saturation, and a phosphate linkage at the *sn*-3 position (Figure 1).^3^ Compared with related phospholipids PC and PE, the distinguishing feature of PS is the covalent attachment of serine to the phosphate, giving PS a net negative charge on the head group. Like other glycerophospholipids, PS is synthesized at specialized intracellular structures called mitochondrial-associated membranes (MAMs), structural and functional domains located between the mitochondria and endoplasmic reticulum (ER) that contain enzymes involved in calcium and innate immune signaling, and phospholipid biosynthesis.^4^ In higher mammals, PS synthesis occurs by two homologous enzymes, phosphatidylserine synthase 1 (PTDSS1) and PTDSS2, both localized in MAMs that appear to have partially redundant activity. Although knockout of either enzyme in mice have unremarkable phenotypes, double PTDSS1/PTDSS2 knockout mice fail to produce PS and is embryonically lethal.^5, 6^ In contrast, yeast deficient in PTDSS (encoded by a single *CHO1* gene) are able to survive when grown on high concentrations of ethanolamine,^7^ suggesting that PS is an essential membrane lipid in higher metazoans. Interestingly, genetic linkage analysis suggest that rare sporadic dominant gain-of-function mutations in PTDSS1 occur in patients with Lenz-Majewski syndrome, biochemically characterized by increased PS in their membranes, and phenotypically by multiple congenital abnormalities of generalized craniotubular hyperostosis.^8^

Following biosynthesis, PS is transported from MAMs to the plasma membrane by carrier proteins where it is actively maintained on the inner leaflet of the membrane by several complementary enzymes. Flippases and Floppases translocate phospholipids from the outer to the inner surface and from the inner to the outer surface, respectively. Scramblases collapse membrane asymmetry thereby randomizing all phospholipid species between leaflets, which in the context of PS biology, effectively increases the accumulation of PS on the external side of the membrane.^3^

Physiologically, the intracellular deposition of PS has structural and biochemical importance.^8^ The net negative charge of PS contributes structurally to membrane curvature and fluidity, and the electrostatic charge provides a docking site for proteins with poly-cationic domains such as C2 and Gla domains.^9^ Indeed, a number of important intracellular proteins require PS for proper localization and/or activation. Such proteins include the E3 ubiquitin-ligase NEDD4, protein kinase C isoforms, several phospholipase C and D isoforms, phosphatase, and tenson homology-deleted on chromosome 10, as well as a number of synaptotagmin isoforms that are required for vesicle trafficking and fusion. In addition, several members of the annexin family of proteins, which have essential roles in membrane–cytoskeletal anchoring and membrane trafficking, bind PS.^9^ Upon loss of membrane asymmetry, PS translocates across the bilayer and interacts with a new set of extracellular serum proteins and PS receptors that trigger an array of biochemical and immunological responses.

Although PS externalization is clearly one of the emblematic signals that tags cells for efferocytosis, PS is also externalized on activated platelets during coagulation and platelet aggregation,^10^ on viable monocytes,^11^ on the surface of mature macrophages,^12^ on myocytes during myoblast fusion,^13^ on nuclei expelled from reticulocytes,^14^ on activated B cells,^15^ on tumor cells,^16^ on extracellular vesicles derived from cancer cells,^17^ and on the surface of exosomes derived from tumors, platelets and dendritic cells (DCs).^18^ However, PS exposure on viable cells does not induce phagocytosis as both amateur and professional phagocytes are able to distinguish between living and apoptotic PS-exposing cells.

## Mechanisms of PS Externalization during cell Stress and Apoptosis

Although the biochemical landscape for PS externalization is still incomplete, recent progress in this area has emerged following the cloning and characterization of two novel scramblases; transmembrane protein 16F (TMEM16F)^19^ and Xkr8 (*ced-8*),^20^ that externalize PS by distinct regulatory mechanisms. TMEM16F is an eight transmembrane domain receptor with aminophospholipid scramblase activity that is critical for calcium-dependent externalization of PS in activated platelets. The importance of TMEM16F in platelet activation was obtained from knockout studies showing that loss of function impairs calcium-dependent PS scramblase activity. This resulted in the inability of platelets to recruit and activate clotting factors with PS-binding Gla domains that include factor V, factor X, and prothrombin.^21^ Loss-of-function mutation in TMEM16F is associated with Scott's syndrome, a rare bleeding disorder characterized by defects in calcium-dependent phospholipid scrambling, suggesting that it is the predominant scramblase for externalizing PS in platelets.^19, 21^ Other members of the TMEM16 family, that include 16C, 16D, 16F, 16G, and 16J have been shown to scramble PS, although it awaits clarification in which cell types, and by what activation signals, these scramblases function.^22^

More recently, a second scramblase, Xkr8, was shown to cause PS externalization in cells dying by apoptosis. In contrast to TMEM16F, Xkr8 is not activated by Ca^2+^, but via a caspase 3/7-dependent pathway. In fact, Xkr8 scramblase activity is very low in living cells, but is activated during apoptosis through a conserved Asp-Glu-Val-Asp-Gly caspase 3/7-cleavage site motif located at its C termini that releases an inhibitory sequence thereby activating scramblase activity.^20^ Interestingly, Xkr8 is a mammalian homolog of the CED8 in *Caenorhabditis elegans*.^23^ Mutated CED8 leads to a characteristic defect in efferocytosis suggesting evolutionary conservation of PS externalization pathways in apoptosis. Other members of the Xkr family have been defined, including Xkr4 and Xkr9 that are also activated by caspases, for example, caspase 3.^24^ Unlike Xkr8 that is ubiquitously expressed, Xkr4 and Xkr9 have tissue-specific inducible expression patterns. This suggests that PS externalization might be dynamically regulated by specific signaling pathways that impact the expression of Xkr4 or Xkr9.

## Not all Externalized PS is Functionally Equivalent

**Figure 2:**
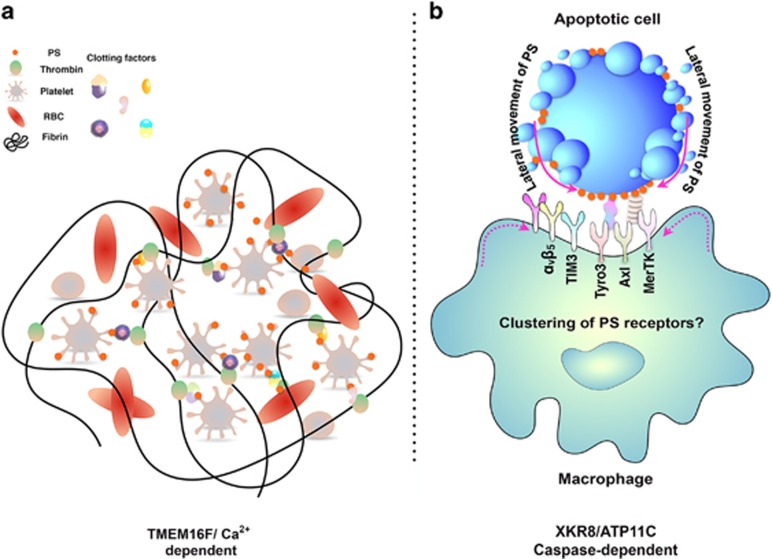
Models for the different forms of PS externalization: As noted in the text, PS can be externalized under a variety of physiological and patho-physiological conditions that include platelet activation (**a**) and caspase-dependent apoptosis (**b**). (**a**) Activated platelets promote a Ca^2+^-TMEM16-mediated externalization of PS that serves as a nucleation scaffold for the recruitment of hemostasis factors that initiate blood clotting (indicated by the solid black line in **a**). (**b**) Apoptotic cells externalize PS via the caspase 3/7-mediated cleavage of Xkr8 that serves as an eat-me signal for various PS receptors (TAMs, TIMs, and *α*v*β*5 and *α*v*β*3 integrins). Recent studies suggest that during apoptosis, the surface density of the PS may reach a critical threshold that clusters and activates PS receptors. Why PS externalized on apoptotic cells (Xkr8-dependent) serves as a signal for efferocytosis, while PS expressed on stressed and activated cells (TMEM16-dependent) has not been completely elucidated

The discussion above highlights an important conceptual idea that different PS scramblases react to distinct upstream signals to externalize PS. Adding complexity to PS biology, it is now apparent that not all externalized PS is functionally equivalent. In the above-mentioned scenario for Xkr8 and TMEM16 that are activated by caspase 3 and Ca^2+^, respectively, only the former serves as an eat-me signal for PS receptors and efferocytosis. Indeed, when a mutant TMEM16F was introduced into a mouse lymphoma cell (W3-Ildm) to achieve constitutive PS exposure, PS-positive tumor cells (assessed as annexin V positive) were not engulfed by professional DCs, and only became phagocytosed after activation of caspase 3 and Xkr8 with Fas antibody.^25^ Thus, the PS externalized by TMEM16 does not provide an eat-me signal, but is sufficient to provide an electrostatic charge to recruit clotting factors via the interactions of their Ca^2+^-dependent Gla domains. Moreover, the Ca^2+^-stimulated PS externalization induced by TMEM16F is rapid (within minutes) and reversible upon restoration of Ca^2+^ homeostasis,^22^ while Xkr8-mediated PS exposure is slow (within hours) and irreversible (Figure 2).

With respect to the externalization of PS by Xkr8 during apoptosis, recent evidence suggests that stable and irreversible PS externalization is achieved by a dynamic interplay between Xkr8 and ATPase, class VI, type 11C (ATP11C), a member of the P4-type ATPase family of flipases that redirects PS from the outer membrane leaflet back to the inner leaflet.^26^ Similar to Xkr8, ATP11C contains a caspase cleavage site, but unlike Xkr8 that is activated by caspase cleavage, ATP11C is inactivated by the same process and prevents return of PS to the inner leaflet. Conversely, when cells express ATP11C with a mutated caspase recognition site, cellular flipase activity remains high, and cells expressing mutant ATP11C do not sustain PS externalization or retain their ability to be engulfed. In the non-apoptotic context, a high Ca^2+^ concentration activates TMEM16, but does not inactivate ATP11C, possibly explaining the reversibility of TMEM16-mediated PS externalization.

The preceding reasoning suggests that a critical concentration or topology of PS may need to be acquired for recognition as an eat-me signal. A possible explanation as to how PS topology or local density might be recognized differently by PS receptors might also be related to the PS clustering activity exerted by Annexin.^27^ The combination of low membrane fluidity and consequent low clustering of PS receptors on the phagocytes' surface due to reduced lateral mobility of the PS molecule may help to distinguish dead/dying from viable PS-exposing cells.^11^ Receptor clustering is often sufficient to activate intracellular signaling cascades. In apoptotic cells the cytoskeleton and the focal adhesion molecules are early targets of caspases. After death receptor stimulation, active caspase 8 immediately translocates to plectin, a major cytoskeletal cross-linking protein and quantitatively cleaves it at Asp 2395.^28^ The resulting weakening of the cytoskeleton increases the lateral mobility of PS and might consequently enable cooperative binding of PS ligands or receptors. Furthermore, there is evidence that lipid rafts and PS are mutually exclusive on the membranes of apoptotic cells in contrast to viable and activated cells.^29^ This suggests that there may be different topologies of PS arranged on the surfaces of apoptotic *versus* viable cells that engage receptors in distinct ways. Indeed, recent studies examining the effects of ligand-density on the activation of AXL receptor tyrosine kinase (Axl; a PS receptor) support this idea, in which it was concluded that the specific sensing of ligand spatial distribution is a critical feature for PS-dependent (Axl) receptor activation.^30^

Although the preceding sections have focused on the interplay between scramblases, flipases, and PS externalization, other enzymes and pathways have been implicated in PS externalization including the ATP-binding cassette (ABC) transporter ABC1^31^ and Tat1.^32^ Moreover, studies by Lee *et al.*^32^ suggest that increases in bidirectional membrane trafficking results in PS externalization. In their model, PS is externalized by a two-step process whereby internalization of plasma membrane into cytoplasmic vesicles occurs as cells shrink during apoptosis. This is followed by Ca^2+^-dependent trafficking of PS-positive vesicles back to the cell surface.^32^ Whether these specialized forms of PS externalization lead to diverse depots of PS on the membrane is not clear, although the recent development of high-resolution fluorescent probes, such as Disciodin-C2, and GFP-LactadherinC2, should make it more feasible to visualize PS in discrete subcellular membrane domains and topologies.^33^

The realization that not all externalized PS has the same biological function also highlights the need to better characterize the nature of the molecular species of PS on the cell surface. Identification of the PS fatty acyl composition, its saturation, length, and oxidative status by mass spectrometry might be instructive in determining whether different externalization itineraries lead to discrete species of PS. With respect to the idea of PS oxidation, in which one or more of the acyl chains has unsaturated and oxidized substitutions, there is some evidence that oxidized PS (oxPS) is a more efficient eat-me signal than the non-oxidized molecule.^34^ Also some PS-binding proteins involved in efferocytosis (i.e., Gas6, milk fat globule-EGF factor 8 protein (MFG-E8), and T-cell immunoglobulin and mucin domain receptor-1 (TIM-1)) appear to bind with higher affinity to oxPS,^34^ which tends to protrude from the planar layers of cell membranes. This is interesting from a mechanistic view, as one of the proposed pathways for PS oxidation involves cytochrome c-dependent PS oxidation, with cytochrome *c* acquiring a gain-of-function peroxidase activity once released from mitochondria.^35^ In this model, cytochrome *c* released during mitochondrial outer membrane permeabilization would serve two interrelated functions. First, as a central component of the apoptosome, and second, to concomitantly catalyze the oxidation of PS to provide an eat-me assurance signal for efferocytosis.^36^ As discussed below, one of the most important future goals will be to assess whether all forms of externalized PS are immunosuppressive.

## Immunological Consequences of PS; Homeostasis, Autoimmunity, and Cancer

The externalization of PS on apoptotic cells serves as a pre-eminent eat-me signal for efferocytosis and allows the controlled elimination of damaged, infected, activated, or senescent cells that would otherwise release potentially harmful cellular contents. The translocation of phospholipids in cellular membranes, for example, PS exposure on the cell surface and cardiolipin translocation within the mitochondrial membranes, are key events in the initial phases of apoptosis and correlate with other major hallmarks of dying cells that include plasma membrane blebbing, cell shrinkage, loss of mitochondrial membrane potential, caspase activation, chromatin condensation, DNA fragmentation, and cytoskeleton remodeling. Collectively, these events are genetically programmed, and are characterized by non-inflammatory and non-immunogenic outcomes that maintain tolerance. Indeed, it has been known for almost two decades that apoptotic cells are potently immunosuppressive.^37^ In contrast, if the clearance of apoptotic cells fails, they may enter the stage of secondary necrosis, a condition involved in the etiology^38^ and pathology^39^ of chronic inflammatory autoimmune diseases. It is also known that post-apoptotic remnants in the germinal centers of lymph nodes can serve as selecting antigens for B cells that have acquired auto-reactivity during the process of somatic mutation.^38^ IgG auto-antibodies recognizing secondary necrotic cells (SNEC) or apoptotic cell-binding ligands are able to shift silent clearance toward inflammation.^40^

At the tissue and systemic level, the rapid non-inflammatory and non-immunogenic clearance of apoptotic cells involves at least three kinds of interrelated pathways that signal; (i) ‘find me', (ii) ‘eat me', and (iii) ‘tolerate me'. With respect to ‘find me' or attraction signals, apoptotic cells actively release chemo-attractants recruiting phagocytes to the site of cell death. The best understood of these factors involve phospholipids such as lysophosphatidylcholine and sphingosine-1-phosphate as well as other mediators (e.g., nucleoside triphosphate with purinergic receptor Y, CX3CL1/fractalkine, endothelial monocyte-activating polypeptide II, and dimeric ribosomal protein S19 with G-protein coupled receptor CD88 and thrombospondin-1 (reviewed in ref. 41)). At the same time, secreted ‘stay away signals' that repel neutrophils, limit the immunogenic damage caused by degranulation.^42^ Together, these signals ensure that a phagocytic system is available in the neighborhood of dying cells. Indeed, all of the major phagocytic cell types; that is, macrophages, DCs, Kupffer cells, microglia, and alveolar macrophages have receptors for apoptotic find-me signals, ensuring that secondary necrosis, and the ensuing immunogenic outcomes resulting from the rupture of the plasma membrane, is minimized.

PS is one of the primary apoptotic cell ligands that provides eat-me signals to phagocytes. Upon recruitment, phagocytes recognize PS directly or indirectly through cell–cell interactions mediated by specific bridging or adapter molecules recruited to the surfaces of dying cells. Macrophages recognize additional abnormal cell characteristics such as elevated lateral mobility of PS^11^ or modifications of the glycocalyx.^43^ These interactions initiate signaling pathways that rearrange the actin cytoskeleton thereby enabling the engulfment of apoptotic cells.^44^

Finally, the rapid and effective removal of apoptotic cells by phagocytes is crucial for prevention of an undesirable inflammatory response and maintenance of an anti-inflammatory status during homeostasis (‘tolerogenic signals'), a term that has sometimes been called silent apoptosis to convey immune downregulation. In contrast to the uptake of pathogens or FcR-mediated phagocytosis, engulfment of apoptotic cells does not induce inflammatory cytokine production. Instead, engulfed apoptotic cells induce the secretion of the anti-inflammatory cytokine interleukin-10 (IL-10) and TGF-*β* and simultaneously decrease the secretion of the inflammatory cytokines TNF-*α*, IL-1*β*, and IL-12.^37, 45^ Moreover, *in vitro* experiments have shown that the production of TGF-*β*, considered a central player in the anti-inflammatory responses of phagocytes, is increased following efferocytosis. Indeed, phagocytes that engulf PLB-985 cells, human monomyelocytes that do not express PS during apoptosis, fail to produce TGF-*β*, whereas incubation of the phagocytes with PS liposomes, or PS directly transferred onto the PLB-985 surface membranes, restored TGF-*β* secretion.^45^ This indicates that PS functions as an immune-suppressing mediator during the clearance of apoptotic cells.

## Consequences of a Failure in Apoptotic Cell Clearance

As noted above, the silent clearance of apoptotic cells is carried out by a reliable phagocytic system that allows rapid recognition and removal in a non-immunogenic and non-inflammatory manner. However, this system, at times, can fail. If clearance is impaired, apoptotic cells can undergo secondary necrosis and cause the release of pro-inflammatory cytokines by phagocytes.^40^ SNEC, characterized by the loss of membrane integrity, release large amounts of modified intracellular and intranuclear macromolecules as well as ions into the surrounding interstitium. In this case, apoptotic cells gain inflammatory potential similar to certain primary necrotic cells increasing the possibility that an immune response can be developed against these neo-epitopes.^44^

Multiple studies support a direct link between a failure of apoptotic cell clearance and the development of the chronic autoimmune disease systemic lupus erythematosus (SLE).^38, 46^ First, as reported almost 20 years ago, macrophages isolated from patients with SLE display a reduced capacity for phagocytosis of apoptotic cell remnants *in vitro*.^46^ Second, in lymph node sections from some patients with SLE, the number of macrophages containing ingested apoptotic material is decreased.^38^ Third, in the same patients, binding of apoptotic nuclear remnants to follicular DCs was observed. This could contribute to the etiology of autoimmunity by supplying survival signals to B cells that have accidentally developed auto-reactivity against nuclear and apoptosis-related autoantigens during somatic diversification, a process that randomly inserts mutations into the variable region of IgG.^38^ Taken together, these observations provide evidence for clearance deficiency as one of the etiological causes of SLE. Furthermore, impaired clearance of apoptotic cells leads to the secretion of anti-nuclear antibodies from auto-reactive B cells. These antibodies form immune complexes with nucleic acid containing apoptotic cell remnants that are cleared by peripheral blood monocytes, macrophages and DCs through Fcγ-R-mediated phagocytosis. Upon Fcγ-R clustering, vast amounts of inflammatory cytokines^47^ are released leading to chronic inflammatory disease and ultimately to multiple organ damage.^40^

## PS Receptors, Efferocytosis, and Surveyors of Immune Homeostasis

During the past decade, great strides have been gained in the identification and characterization of PS receptors and PS-binding opsonins (endogenous proteins that bridge PS to efferocytes). This has increased our understanding of how apoptotic cells are removed in tissues. There is now definitive evidence from knockout mice demonstrating that effective PS-dependent clearance protects organisms from secondary necrosis. Mice deficient in individual PS receptors, for example, Mertk, TIM-3, SCARF-1, as well as PS opsonizing proteins MFG-E8, C1q, or Protein S, exhibit a failure in the clearance of apoptotic cells and the subsequent elevation in pro-inflammatory cytokines such as IL-1*β* and TNF-*α*.^48^ These observations convincingly link PS recognition by PS receptors with removal of immunogenic debris that prevents autoimmunity (discussed above).

With over a dozen PS receptors and opsonins that span a wide range of gene families, there are likely to be overlapping and non-overlapping mechanisms whereby PS receptors can invoke immune suppression and tolerance. These effects could be passive and indirect, by ensuring efficient efferocytosis that safeguards against secondary necrosis and the release of signals associated with danger-associated molecular patterns that activate Toll-like receptors (TLRs). Conversely, PS receptors can also function as direct inhibitory receptors that dampen inflammation and/or induce immune suppression. Among this latter group, the inhibitory TAM and TIM receptors are among the best-studied PS receptors.

**Figure 3:**
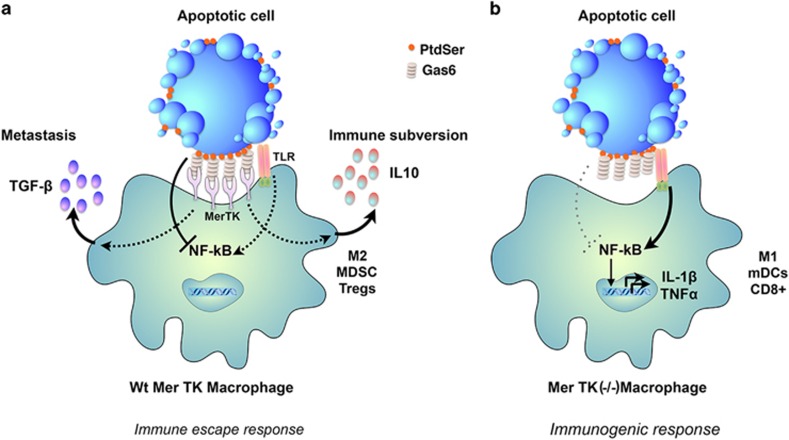
The PS receptor Mertk acts as an inhibitory receptor to promote homeostasis and tissue tolerance: Mertk, a member of the TAM family of PS receptors, interacts with externalized PS on apoptotic cells via its bridging molecule Gas6 to drive efferocytosis and tissue tolerance. Once engaged, Mertk transmits an inhibitory signal that inhibits NF-kB and the production of inflammatory cytokines from TLRs. Efficient efferocytosis also produces the production of tolerogenic factors such as IL-10 and TGF-*β* that tolerize the local microenvironment in favor of M2 macrophages, immature DCs, and Tregs. When Mertk is targeted by knockout, or inhibited by therapeutics, TLR-induced activation of inflammatory cytokines proceeds unabated in the absence of dampening signals, leading to an immunogenic environment characterized by the production of M1 macrophages, antigen presenting mature DCs and CD8^+^ T cells as discussed throughout the text

The TAM receptor tyrosine kinases (Tyro3, Axl, and Mertk) and their cognate ligands, Gas6 and Pros1, are essential in the resolution of inflammation, and have direct anti-inflammatory activity that suppresses nuclear factor*-κ*B (NF-*κ*B) and inflammatory cytokines. In the case of Mertk, which is abundantly expressed on M2 macrophages and bone marrow-derived DCs (BMDCs), the tyrosine kinase transmits a PS-dependent inhibitory signal that prevents LPS-inducible phosphorylation of I*κ*B kinase, degradation of I*κ*B, and the activation of NF-*κ*B.^49^ This effect is Mertk-specific, as BMDCs from Mertk^−^^/−^ mice fail to show inhibition of NF-*κ*B activation. These effects on NF-*κ*B inhibition are separable from those on efferocytosis,^50^ an observation consistent with previous findings that binding of apoptotic cells to the surface of phagocytes is sufficient for the downregulation of inflammatory cytokines^51^ (Figure 3).

Studies with the related TAM receptor Axl provides further mechanistic insight into how PS receptors transmit immune inhibitory signals. Unlike Mertk that is constitutively expressed on macrophages and DCs, under basal conditions the expression of Axl in DCs is low, but is significantly upregulated as a consequence of TLR engagement to resolve and break inflammation in anticipation of the end of an inflammatory cycle.^52^ At a mechanistic level, Gas6-induced activation of Axl suppresses TLR and type I interferon (IFN) receptor JAK-STAT signaling by upregulating the expression of SOCS1 and SOCS3, thereby turning off the expression of inflammatory cytokines including TNF-*α*, IL-1*β*, and IFN-*α*.^52^ Teleologically, a similar mechanism appears to operate in antigen-activated T cells, whereby activated T cells upregulate Pros1 and drive a PS/Tyro3-dependent inhibitory signal.^53^ Pros1^−/−^ mice fail to suppress T-cell activation, suggesting this mechanism is in place to prevent T-cell over-activation.

Similar to the TAMs, the TIMs comprise another class of PS receptors that directly relay inhibitory signals from PS.^54, 55, 56, 57, 58^ In humans, there are three main subtypes of TIM receptors (TIM-1, TIM-3, and TIM-4), each of which has confirmed PS interactions through a conserved N-terminal IgV extracellular domain. Although similar in structure (all are type I membrane receptors), TIM receptors have unique features and are expressed on different cell types. TIM-4 is the only receptor of the family that does not harbor any intracellular tyrosine phosphorylation motifs, suggesting it is a tethering molecule that does not independently transmit a signal.^59^

Characterization of both TIM-1 and TIM-3 modulation of T-cell responses have been described, and a detailed signaling pathway downstream of activation of these receptors is beginning to emerge. Out of the three receptors, TIM-3 has been the most intensely studied with regard to cancer and viral infection, as it is potently immunosuppressive when activated. One active area of research is investigating the role of TIM-3 in T-cell exhaustion, which is a defective T-cell response found in many chronic infections and in cancer.^60^ In a recent study investigating TIM-3 signaling pathways, Rangachari *et al.*^61^ found that HLA-B-associated transcript (Bat3), a chaperone protein known to bind to the intracellular tail of TIM-3, associates with the active domain of Lck. Agonistic antibody ligation of TIM-3 led to the dissociation of Bat3, allowing active Lck to associate with the cytoplasmic tail. These results are consistent with studies showing interactions between TIM-3 and Lck, along with Src family tyrosine kinase Fyn.^62^ The mechanism resembles that of immunoreceptor tyrosine-based inhibition motifs on inhibitory receptors (ITIMs). Other studies suggest more complex actions of TIMs; for example, TIM-3 expressed on tumor-associated DCs suppressed TLR-mediated innate immune responses to nucleic acids by interfering with HMGB1-mediated anti-tumor immune-surveillance mechanisms.^63^ Although these studies provide significant insight into the TIM-3 signaling pathway, there is still much to be elucidated. For example, it is not known how IL-10 is upregulated during efferocytosis, and whether it is driven by engagement of a PS receptor.

## Consequences of Silent Clearance in Viral and Protist Infection, and in Cancer

**Figure 4:**
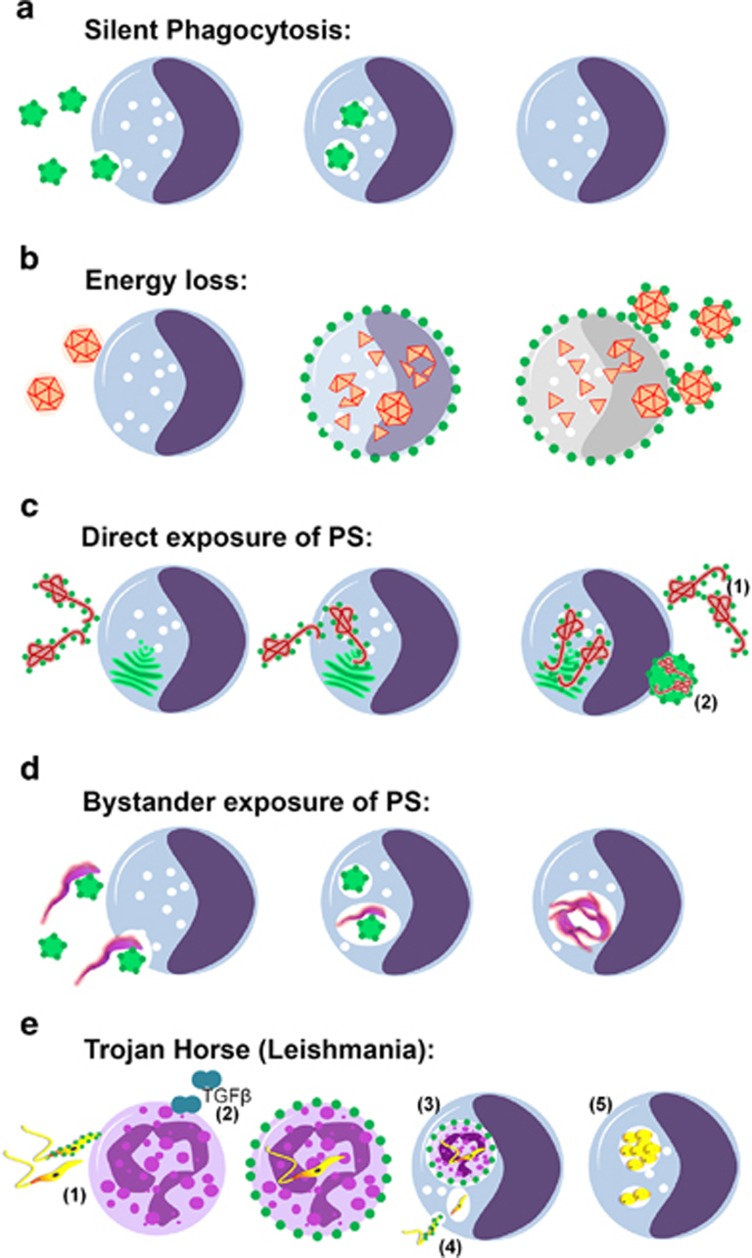
Modalities of PS exposure on infectious agents: (**a**). Silent Phagocytosis Clearance of PS-exposing particles (e.g., apoptotic remnants), via silent phagocytosis of monocytes/macrophages. (**b**). Energy loss Diminished energy reserves cause breakdown of membrane asymmetry and lead to PS exposure on infected monocytes (e.g., HCV). Enveloped Virions budding from infected cells expose PS. (**c**) Direct exposure of PS on HIV-1, Ebola, Variola and other highly pathogenic enveloped viruses. (1) Replication and budding from PS-rich surfaces (e.g., golgi apparatus, endoplasmic reticulum) allows PS exposure on newborn virions. (2) Clusters of Enteroviruses are packed and released non-lytically in PS-exposing lipid vesicles elevating their infectivity. (**d**) Bystander exposure of PS (*Trypanosoma brucei*) *T. brucei* evolutive forms do not expose PS. Parasites are engulfed as bystanders together with PS-exposing apoptotic cell remnants. (**e**) Trojan horse (Leishmania) (1) Upon infection of the host PS-exposing Leishmania promastigotes are engulfed primarily by neutrophils. (2) ‘Apoptotic', PS-exposing Leishmania promastigotes induce release of TGF-*β* by neutrophils silencing their leishmanicidal response at the side of the sand-fly bite. (3) Infected PS-exposing Neutrophils are silently phagocytosed by monocytes enabling intracellular replication. Hiding of promastigotes in ‘apoptotic' neutrophils not only delivers viable Leishmania into macrophages but also delays the immune response against the parasite until the first line neutrophilic response is resolved. (4) PS-exposing Leishmania promastigotes can actively invade into monocytes for intracellular replication. (5) Persistent infection and intracellular replication in the amastigote form within professional phagocytes

Although ‘silent clearance' of the apoptotic cells is necessary to maintain homeostasis, in cancer, exposure to radiation, and some parasitic, viral and bacterial infections, the ubiquitous mechanism of non-inflammatory apoptosis might be disadvantageous for the host. Indeed, pathogens involved in the most severe infectious disease utilize PS exposure for silent apoptosis to ensure their own survival. These aspects of PS biology are discussed in the subsequent sections and are described in Figure 4 and Tables 1a and 1b.

## Viruses Employ PS Externalization and Apoptotic Mimicry to Evade Host Responses

As obligate intracellular pathogens, viruses have evolved many elegant strategies that subjugate host cell factors and functions to assure their successful entry and replication. It has recently come to light that viruses use a strategy termed viral apoptotic mimicry to hijack essential apoptotic recognition and clearance mechanisms for their own means. This mechanism, whereby viruses mimic apoptotic debris by concentrating PS within their membranes (enveloped viruses), or cloaking themselves in cell-derived PS-containing vesicles (non-enveloped viruses), is emerging as a common theme used by many virus families to facilitate virus binding, entry, and immune evasion.^64^ Indeed, viral apoptotic mimicry has proven to be a widespread lipid mediated entry mechanism used by several enveloped viruses including: Vaccinia, Pichinde, Cytomegalo, Lassa Fever, Lenti, Dengue, Ebola and Marburg viruses, and non-enveloped viruses:^64, 65^ SV40, Hepatitis A, and Polio^66, 67^ (Tables 1a and 1b). Given the anti-inflammatory nature of apoptotic clearance, it is easy to envision why a virus would evolve to use an apoptotic mimicry strategy. In addition, as professional and non-professional phagocytes are capable of clearing apoptotic debris and there are multiple PS receptors, by using apoptotic mimicry viruses may expand their cell-type specificity and tropism without the need for specific receptor ligands.

For viruses using apoptotic mimicry, the acquisition of envelope PS during virus assembly is critical. Viruses use different means to acquire PS in a process that is largely dependent upon the intracellular compartment in which they replicate. For enveloped viruses this is achieved by budding through intracellular organelles or from the plasma membrane. The luminal leaflet of the ER membrane, for example, is rich in PS^1, 3^ making it an obvious lipid source for viruses looking to incorporate PS into their membranes. Two viruses using apoptotic mimicry, dengue and vaccinia virus, are thought to acquire their PS-rich membranes by budding into the ER lumen.^68^ Human immunodeficiency virus (HIV), which uses PS as cofactor for infection, acquires PS by budding from lipid rafts.^69^ For non-enveloped viruses, PS acquisition relies largely on hijacking of intracellular membranes including multivesicular bodies and autophagy-like organelles that are rich in PS.^70^

Although viral apoptotic mimicry was originally hypothesized to be immune evasion strategy explaining the silent infection employed by hepatitis B virus, this process was first experimentally linked to the induction of poxvirus endocytosis.^71^ The rational being that by mimicking an apoptotic body, poxviruses hijack the indispensable apoptotic clearance machinery of host cells to promote virus internalization.^72^ Since this initial finding, viral apoptotic mimicry has been found to facilitate binding, entry and immune evasion by viruses from 10 different families (reviewed in refs. 64 and 65). For many of these viruses, the PS receptors and/or bridging molecules they engage and the purpose for which they employ apoptotic mimicry has been defined.

As discussed above, apoptotic clearance is intimately linked with the dampening of inflammatory responses. This involves the induction of anti-inflammatory cytokine production, as well as inhibition of inflammatory cytokine secretion and TLR signaling. Thus, in addition to promoting uptake and binding, apoptotic mimicry by viruses potentiates infection by dampening host innate immune responses. An early indication of this comes from a study of pseudotyped lentiviral particles which, when complexed with Tyro3/Axl/Mer (TAM) bridging molecules, Gas6 or Protein S, act as ‘super TAM agonists' that disable host immune responses and facilitate virus spread.^73^ In this study, the authors demonstrate that enhancement of viral infection is associated with TAM-mediated inhibition of type I IFN signaling. They found that BMDCs from TAM knockout mice produced high levels of IFN-*α*, IFN-*β*, and SOCS1 mRNA relative to WT BMDCs when challenged with PS-containing pseudotyped lentiviral particles. In addition, inclusion of anti-IFN *α*/*β* antibodies restored lentiviral infection in TAM triple KO BMDCs nearly to the level of infection seen in WT cells. These data suggest that enhancement of viral infection promoted by TAM engagement is primarily due to inhibition of the antiviral type I IFN response. In the case of vaccinia, *in vivo* infection with vaccinia virus results in the induction of anti-inflammatory cytokines including TGF-*β* and IL-10, prevention of macrophage infiltration, and inhibition of T-cell maturation.^74^

The prominent utility of apoptotic mimicry, including from highly pathogenic viruses such as ebola and dengue (Table 1a) begs the question – can viral apoptotic mimicry be targeted therapeutically? Several lines of evidence suggest this may be possible.^75, 76^ Treatment of animals infected with Pichinde virus with PS-targeting antibodies protected animals from lethal viral infection *in viv*o.^76^ *In vitro* studies demonstrated that PS-targeting antibodies potently inhibit HIV-1 infection of peripheral blood mononuclear cells by facilitating upregulation of chemokines known to block receptors utilized by HIV-1 for cell entry.^77^ In addition, *in vitro* studies have shown that PS-targeting antibodies bind several enveloped viruses and enveloped virus-infected cells, including ebola, influenza, and vaccinia. Recent studies showing that PS is involved in non-enveloped virus infections^66^ suggest that PS-targeting antibodies could also be employed to treat such infections. A recent report by Shibata *et al.*^78^ showed that Axl-targeting antibodies attenuate influenza and RSV lethality *in vivo* through modulation of innate immune responses. These data suggest that several small compound inhibitors directed against Axl and Mer that are in various stages of pre-clinical/clinical development as cancer therapeutics might also be therapeutically efficacious in viral infections. Collectively, these results suggest that inhibition of PS-mediated host responses by antibody targeting of PS could provide a new class of antiviral therapeutics.

## Bacteria Exploit Host Cell efferocytosis and use PS Cloaking to Facilitate Cell-to-Cell Spread

Similar to viruses, bacteria also take advantage of efferocytosis in host cells to promote cell-to-cell spread. This mechanism has recently been described in *Listeria monocytogenes.*^79^ *L. monocytogenes* are extruded from the cell membrane of infected macrophages packaged in PS-coated vesicles, which then interact with TIM-4 on other macrophages to facilitate their uptake and cell-to-cell spread. Other bacterial pathogens that also appear to exploit efferocytosis and/or PS cloaking have been identified, such as *Mycobacterium tuberculosis*, *M. avium*, *M. marinum*, and *Chlamydia*. Interestingly, efferocytosis appears to be an effective mechanism by which the host can keep *Mycobacterium* species under control,^80^ highlighting the fine balance between host cell defense and pathogen dispersal mechanisms. Whether or not other bacterial species utilize efferocytosis and PS cloaking generally to their advantage remains to be seen.

## Protozoan Parasites Utilize PS and Apoptotic Mimicry to Evade Host Immune Responses

**Figure 5:**
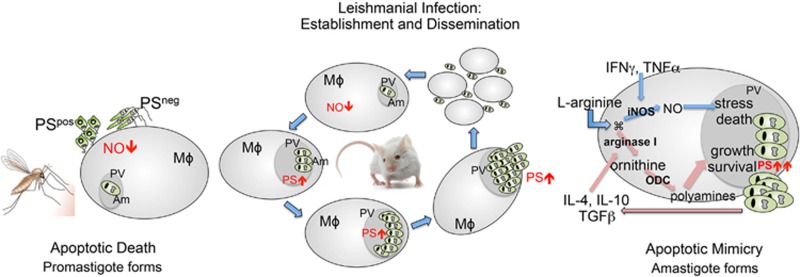
Apoptotic death versus apoptotic mimicry during leishmanial infection and establishment. Metacyclic promastigotes accumulate in the sand-fly hindgut. The infective inoculum contains live parasites together with morphologically and biochemically characterized apoptotic parasites. The presence of apoptotic and viable parasites is necessary for the establishment of the infection. Live parasites infect host cells, whereas dead parasites downregulate the production of nitric oxide (left panel). In the mammalian host, amastigote forms disseminate the disease and expose PS at their surface without any other phenotype of apoptotic death (center panel). PS recognition by the macrophage leads to an active anti-inflammatory response, mainly characterized by TGF-*β* and IL-10 production. This generates a feedback effect leading to increased macrophage deactivation and parasite proliferation. Susceptible mice strains upregulate PS exposure on intracellular amastigotes by a mechanism yet to be defined (right panel)

In addition to viruses and bacteria, there is a growing body of evidence that various protozoans also use PS for apoptotic mimicry and immune subversion as a part of their infectious lifecycle. However, the function of PS in protozoan infectivity is complicated by the fact that protozoans can activate a classical programmed cell death (PCD) pathway to externalize PS, but also externalize PS to evade immune surveillance. The regulatory pathways that govern these events are now beginning to be unraveled (Figures 4 and 5).

Apoptotic-like death has been described in three different species of trypanosomatids: *Trypanosoma cruzi*,^81^ *Trypanosoma brucei*,^82^ and *Leishmania amazonensis*,^83^ etiological agents of neglected endemic diseases that include Chagas' disease, African trypanosomiasis and Leishmaniasis, respectively. Following these reports, a large number of descriptions of apoptosis and apoptosis-like cell death in pathogenic and non-pathogenic unicellular organisms were reported.^84^ These different types of cell death were first described as resulting from environmental stresses, suggesting that single cell organisms, like higher metazoans, were programmed for their cellular demise. For example, death of promastigotes of *Leishmania amazonesis*, with apoptotic features, was described upon treatment with the calpain inhibitor, MDL28170.^85^ In addition, promastigote death and PS exposure is inhibited by Z-VAD-FMK in stationary-phase cultures.^86^ Indeed, with the exception of rhizaria, apoptotic markers, including PS externalization, have been observed in unicellular organisms of all major groups of prokaryotes.^84^ Collectively, these studies demonstrated that unicellular organisms could undergo an apoptosis-like cell death program with phenotypic features resembling apoptosis of multicellular organisms (Figure 5).

The apparent conservation of apoptotic cell death machinery in single cell protists raises several important questions. What is the biochemical machinery that executes and controls these events? What is the teleological significance of the selective pressures that shape the evolution of apoptosis-like death in these organisms and what benefit do they have on population dynamics?^87^ Importantly, biochemical analysis of the various components of different types of cell death together with bioinformatics-based comparisons between PCD pathways in the different species of the phylogenetic evolutionary tree have found PCD-related sequences.^88^ The phylogenetic distribution of such sequences indicates that the PCD machinery operating in multicellular organisms had its origin in the early stages of eukaryote evolution, suggesting that death by apoptosis is phylogenetically conserved.^84, 89^

In addition to the PCD in single cell protists, elegant studies in *Leishmania spp*, and *Leishmania amazonensis*, have led to a conceptual distinction between apoptotic death (in which the organism dies) and apoptotic mimicry (in which the organism mimics death to favor infectivity).^90^ In both situations, PS exposure on the surface of the parasite has an important role in host/parasite interaction. In amastigotes, the form responsible for disease dissemination in mammalian hosts, humans included, PS exposure without parasite death, has been described and shown to be modulated by the host in a murine model of the disease.^90, 91^ On the other hand, in promastigotes, a sub-population of metacyclic parasites die, displaying several phenotype markers of apoptotic death, including PS exposure.^90^ Apoptotic promastigote forms can be observed in axenic cultures and in the gut of the sand-fly vector. In each situation PS exposure contributes to parasite infectivity.

Since its description in 2001, apoptotic mimicry, particularly PS exposure, has been implicated in the establishment of various intracellular protozoan infections, including modifying host immune function through the production of IL-10 and TGF-*β*.^91^ The infective inoculum of *Toxoplasma gondii* parasites comprise two different populations of tachyzoite forms, PS-exposing and non-exposing ones, that cooperate to establish infection in a similar way to what happens with *Leishmania* promastigotes.^92^ Interestingly, in this case PS-exposing tachyzoites are responsible for disease dissemination.^92^ The infective trypomastigote forms of *Trypanosoma cruzi*, subvert the inflammatory capacity of macrophages by activating Smad 2 nuclear translocation and inducible NO synthase enzyme degradation in host cells. Unlike the *T. cruzi* amastigote and epimastigote forms, the evolutive form is the only form that is capable of exposing PS.^93^

The molecular mechanism involved in PS exposure in pathogenic trypanosomatids is just beginning to be unraveled. Although it is not yet clear whether Xkr8 and TMEM16 are phylogenetically conserved in these organisms, Campos-Salinas *et al.*^94^ recently described the functionality of a novel ABC transporter in PS externalization in three different species of *Leishmania* spp. A functional defect in this translocase decreased PS exposure in promastigotes^94^ that correlated with the loss of parasites' infectivity in a murine model of experimental leishmaniasis.

The obligate intracellular pathogen Leishmania major survives and multiplies in professional phagocytes. Intriguingly, the infection process of Leishmania is based on two steps, both governed by PS. A mixture of PS^+^ and PS^−^ promastigotes enters the host body at the site of the sand-fly bite/needle injection. (i) Within 1–3 h, PS^+^ or PS^+^ together with PS^−^ promastigotes, but not PS^−^ promastigotes alone, are engulfed silently by neutrophils. These infected neutrophils, undergo apoptosis and expose PS, thus promoting immune evasion (ii) PS^+^-infected neutrophils and their apoptotic PS^+^ remnants recruit professional phagocytes, the preferred host cells for intracellular replication. Silent clearance by circulating monocytes and tissue resident macrophages, allows leishmania promastigotes to enter their replicatory state (amastigotes) within monocytes undetected by immune surveillance^95^ (Figure 4e). Presently, studies employing leishmania are among the best understood models of protist apoptotic mimicry.

## Externalized PS is Dysregulated in the Tumor Microenvironment

**Figure 6:**
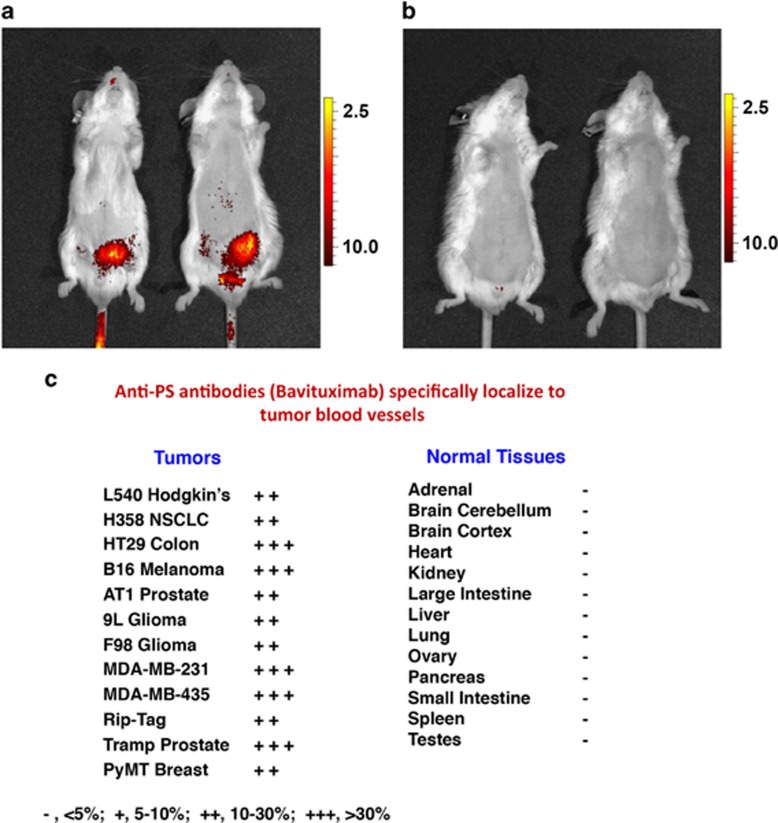
PS targeting antibodies selectively target the tumor microenvironment. Localization of near-infrared (NIR)-labeled Bavituximab F(ab)_2_ to orthotopically implanted PC3 prostate tumor in male SCID mice. Animals were injected with 25 μg NIR-PGN650 (**a**) or NIR-control IgG F(ab')_2_ (**b**). Fluorescent imaging was conducted 24 h following injection of NIR-labeled antibodies. Anti-PS antibodies specifically localize to tumor blood vessels (**c**)

As noted above, the non-immunogenic properties of apoptotic cells can be hijacked by tumor cells to escape immune detection by creation of a local immunosuppressive environment that is defined by the presence of IL-10, TGF-*β*, soluble FAS and FAS-ligand. In addition to the increased burden of apoptotic cells, pro-inflammatory and adaptive immune response are suppressed in the tumor microenvironment by the presence of (i) immature tumor vasculature,^96^ (ii) tumor-derived exosomes,^18^ and (iii) viable tumor cells,^16^ all of which express PS (Figure 6). Moreover, intra-tumoral DCs that bind and ingest PS-expressing cells maintain an immature phenotype preventing the expression of co-stimulatory molecules that are required for optimum functional antigen presentation.^97^ PS exposure on microvesicles (exosomes) derived from patient tumor samples also suppress activation of T-cell responses.^98^ In addition, PS is markedly increased in tumors in response to chemo- and radiotherapy, which further enhances PS-mediated immunosuppression.

## Function of PS Receptors in Cancer Microenvironment

PS receptors, including the TAM and TIM family of receptors, are expressed on infiltrating myeloid-derived cells where they function to promote tissue homeostasis following inflammatory signaling. In the tumor microenvironment these receptors are engaged by PS or PS bridging molecules resulting in the expression of immunosuppressive cytokines and the prevention of a productive anti-tumor immune response. Mertk and Axl are expressed on infiltrating macrophages and DCs, but also frequently expressed on the tumor cells themselves.^99^ This combined effect of PS and PS receptors may provide a ‘perfect storm' that accentuates immune escape. Indeed, elegant experiments by Cook and colleagues showed that transplantation of monocytes from Mertk^−/−^ mice into irradiated tumor-bearing mice support a more favorable anti-tumor response compared to transplantation of wild-type monocytes. This was characterized by decreased levels of IL-10 and increased numbers of tumor-infiltrating lymphocytes, a general feature of improved tumor immunity.^100^ Further studies showed that Mertk-dependent efferocytosis of apoptotic mammary cells by Mertk^+^-infiltrating macrophages during breast involution is associated with TGF-*β* production and increased metastatic frequency of primary breast carcinoma.^101^ As Mertk-dependent efferocytosis requires the vitamin K-dependent PS-binding protein Gas6 for activation, these studies suggest that PS and PS receptors are drivers of both metastatic disease and immune escape. Because warfarin antagonizes GAS6-mediated activation, low dose Warfarin therapy during pregnancy may reduce pregnancy-associated breast cancer progression. This concept is supported by recent studies showing Axl-dependent anti-metastatic activity of warfarin in other solid tumor models, including pancreatic cancer.^102^

## Pre-clinical PS Targeting Agents in Cancer and Infectious Disease; Annexin A5 and mAbs

The above-mentioned dysregulation of PS in the tumor microenvironment suggests that strategies that inhibit PS signaling thereby preventing PS-mediated immune suppression in tumors are attractive. In fact, PS blocking strategies may function akin to immune checkpoint inhibitors, much the same way that blockade of PD-L1 and CTLA4 operate to prevent inhibitory signals in T cells.^103^ Indeed, early pre-clinical studies with Annexin A5 (AnxA5), a natural occurring ligand for PS, support this idea.^104^ Interestingly, the interactions of AnxA5 with apoptotic monocytes proceed in a cooperative manner in the presence of calcium, whereas binding to necrotic as well as viable monocytes does not. As mentioned above, the higher lateral mobility of PS on dying cells may allow binding of a critical density of AnxA5 to saturate and block exposed PS, or it may allow clustering of PS molecules that enhance their signaling capabilities.

Systemic administration of AnxA5 or other PS ligands may slow tumor progression by blocking the tumor-supportive properties of apoptotic cells and tumor-derived microvesicles.^105^ In combination with radio- or chemotherapy, AnxA5 could be used as a natural adjuvant to increase the immunogenicity of dying tumor cells thereby promote an anti-tumor immune response.^106^ This may be especially helpful in targeting cancer cells that have resisted therapy and are thus prone to recurrence and metastases. Incubation of apoptotic cells with AnxA5 prior to immunization has been shown to significantly increase the immunogenicity of these cells.^107^ Thus, the disruption of the PS-derived signals of apoptotic tumor cells by AnxA5 may trigger a pro-inflammatory response contributing to a specific immune reaction against the tumor cells. Interestingly, AnxA5 decreased apoptotic cell uptake by peritoneal macrophages, increased their uptake by DCs, and heightened the immunogenicity of irradiated lymphoma cells *in vivo*.^97, 108, 109^ The fact that AnxA5 has been shown to serve as an adjuvant for apoptotic tumor cells by blocking PS-dependent signals in phagocytes,^97^ supports the further development of annexin proteins as PS antagonists.

With respect to the role of AnxA5 in infectious diseases, the infectivity of HIV-1 for human macrophages is decreased in the presence of AnxA5.^106^ Moreover, PS and a non-phospholipid component of the Hepatitis B virus (HBV) envelope are involved in AnxA5 binding and HBV infection.^110^ The disruption by AnxA5 of PS-mediated signals might be utilized for therapeutic interventions in a multitude of infectious diseases in which apoptotic mimicry causes silent phagocytosis of – and tolerance to – the pathogenic agent (see above).

## PS-targeting Antibodies

**Figure 7:**
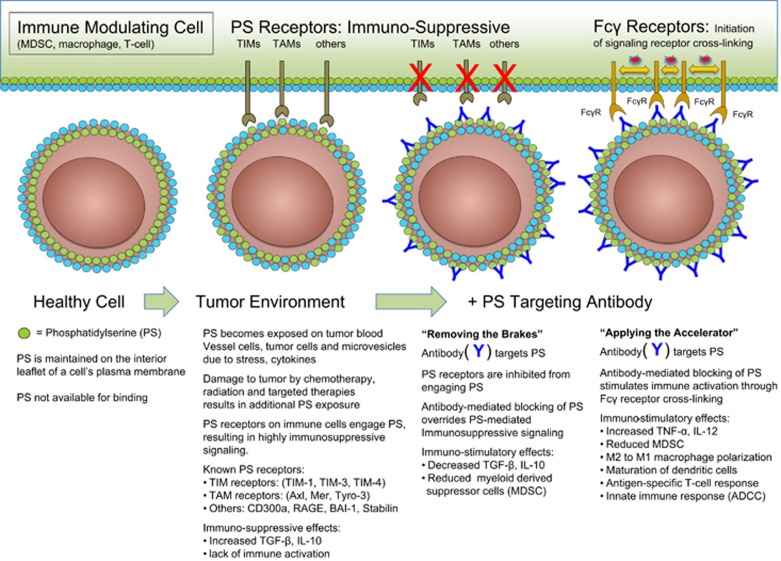
Antibody-mediated blockade of the PS signaling pathway in the tumor microenvironment. As described in the text, PS is highly dysregulated in the tumor microenvironment by the combined action of a oxidative stress and immature tumor vasculature, the secretion of PS-positive tumor exosomes, and the high apoptotic index of proliferating tumors. PS-targeting antibodies are thought to bind externalized PS and interfere with the inhibitory functions of PS in the tumor microenvironment by inhibiting PS binding to PS receptors and by Fc*γ*-mediated ADCC. The net effect is to activate immunogenic signals in the tumor microenvironment

A panel of PS-targeting antibodies that bind to PS with high affinity, either directly or when complexed to the serum protein *β*2-glycoprotein I (*β*2GP1), were first developed by Phil Thorpe's laboratory.^111, 112^ Many of these antibodies bind to exposed PS by cross-linking two molecules of *β*2GP1 thus stabilizing its interaction with externalized PS. Pre-clinical tumor studies showed that the PS-targeting antibodies 3G4, 2aG4 and chimeric 1N11 (mch1N11) localize to PS-expressing tumors and tumor blood vessel endothelial cells, eliciting strong anti-tumor effects when combined with chemo- or radiotherapy (Figure 7 and Table 2). As PS exposure on tumor vasculature was found to be an exquisitely selective marker of endothelial cells in the tumor microenvironment,^96^ these antibodies can be used as vascular targeting agents. However, rather than being limited to the use as delivery vehicles, the antibodies were also found to have anti-tumor activity.^91^ Furthermore, antibody-mediated blockade of PS signaling dramatically enhanced the activity of standard therapies in multiple pre-clinical tumor models.^113, 114^ Evaluation of the tumor vasculature after antibody-mediated PS blockade revealed accumulation of macrophages around tumor blood vessels and subsequent vascular destruction akin to antibody-dependent cellular cytotoxicity^114^ (Figure 7).

Further exploration revealed that irradiation in combination with antibody-mediated PS targeting resulted in long-term durable responses in a syngeneic rat brain tumor model. These results were particularly striking as long-term responders were immune to rechallenge with the same tumor cells implanted contralaterally in the brain.^114^ Subsequent studies have demonstrated in pre-clinical models of prostate cancer that antibody-mediated PS blockade reprograms the innate immune system to promote anti-tumor responses. Additional pre-clinical studies have further delineated multiple measurements of immune activation in the tumor microenvironment mediated by 2aG4, including the increased production of inflammatory cytokines, reduction of immunosuppressive myeloid-derived suppressor cells (MDSCs), and an increase in tumor-fighting M1 macrophages and mature DCs that lead to the induction of tumor-specific cytotoxic T cells.^115^ Recently, studies in immune competent mice bearing breast cancer or melanoma revealed that the combination of PS-targeting (mch1N11) and immune checkpoint antibodies (anti-PD-1 and anti-CTLA-4) showed greater anti-tumor effects than single agent therapy. Combination therapy enhanced the levels of CD4^+^ and CD8^+^ tumor-infiltrating lymphocytes, elevated the fraction of cells expressing the pro-inflammatory cytokines IL-2, IFN-*γ*, and TNF-*α* and increased the ratio of CD8 T cells to MDSCs and Tregs in tumors. Similar changes in immune cell profile were observed in splenocytes. Taken together, these data show that antibody-mediated PS blockade enhances the anti-tumor efficacy of immune checkpoint inhibition.

## Clinical PS-Targeting: Bavituximab

The above-mentioned pre-clinical studies supported the development of a PS-targeting antibody, bavituximab, which is currently being assessed in multiple clinical trials^116, 117, 118^ and planned clinical studies to evaluate the therapeutic efficacy of bavituximab in combination with an anti-PD-L1 antibody for the treatment of solid tumors. Bavituximab is a chimeric monoclonal antibody constructed from the v region (F_v_) of the murine antibody 3G4, used in extensive pre-clinical studies, joined to the c region (F_c_) of a human IgG1. Bavituximab, like 3G4, binds to PS via *β*2GPI. The antibody has been administered to over 700 patients in clinical trials evaluating the antibody as monotherapy and in various combination regimens in patients with multiple cancers, chronic hepatitis C virus and HIV infection. To date, studies have shown promising signs of activity and an acceptable safety profile (Table 2). Moreover, bavituximab has been evaluated in several investigator-sponsored trials that include Her2-negative breast cancer, non-small cell lung cancer (NSCLC), hepatocellular carcinoma, rectal carcinoma, advanced melanoma, and castrate-resistant prostate cancer. Finally, bavituximab is currently being evaluated in SUNRISE (‘**S**timulating Imm**U**ne Respo**N**se th**R**ough Bav**I**tuximab in a Pha**SE** III Lung Cancer Study'), a global randomized, double-blind, placebo-controlled registration trial sponsored by Peregrine Pharmaceuticals (Tustin, California, USA). The SUNRISE trial will assess bavituximab plus docetaxel *versus* placebo plus docetaxel in 582 patients with previously treated locally advanced or metastatic NSCLC.

## Future Perspectives

Although initially characterized as one of the emblematic signals associated with apoptosis, externalized PS on the surface of the apoptotic cell provides a global immunosuppressive signal that dampens local and systemic immunity. This pathway appears to be an evolutionarily conserved mechanism of (higher) metazoans to protect from autoimmune complications when routinely disposing of dying host cells. These signals appear to be breached in autoimmunity, and subverted in viral and protist infection as apoptotic mimics. The multitude of genetically diverse pathogens that have been shown to similarly hijack this fundamental immunosuppressive pathway support a broad ‘apoptotic mimicry' paradigm of pathogenesis and the hypothesis that evolution may have selected for pathogens that steer immune modulating cells toward such ‘survivable' behavior. Moreover, PS appears to be universally dysregulated in cancers, and along with the upregulation of PS receptors, provide potent immunosuppression in the tumor microenvironment. The large amount of evidence obtained with AnxA5 and PS-targeting antibodies supports the notion that PS is a fundamental immune checkpoint akin to or upstream of the CTLA4 and PD-1/PD-L1 checkpoints. Late-stage clinical trials evaluating the PS-targeting antibody, bavituximab, are in progress in multiple oncology indications, while agents targeting PS receptors are in various stages of pre-clinical and clinical development.

## Figures and Tables

**Table 1a:** List of viruses that employ apoptotic mimicry

**Virus family**	**Role of apoptotic mimicry**	**Phosphatidylserine receptors**
*Enveloped viruses*		
Alphavirus (CHIKV, RRV, SINV, EEEV)	Binding, endocytosis, and infection	TIM-1, TIM-4, AXL, Integrins (MFG-E8 binding), CD300A
Arenavirus (LASV, AMAV, TCRV, LCMV, Pichinde)	Binding, endocytosis and infection	AXL, Tyro3, TIM-1
Baculovirus	Binding, endocytosis and infection	AXL, TIM-1
Filoviriruses (EBOV, MARV)	Binding, endocytosis, infection and Immune evasion	AXL, Tyro3, TIM-1 TIM-4
Flavivirus (DENV, WNV, YFV)	Binding, endocytosis, infection and Immune evasion	TIM-1, TIM-3, TIM-4, AXL, Tyro3
Poxvirus (VACV MV and EV)	Signaling, endocytosis and infection	AXL
Rhabdovirus (VSV)	Binding, endocytosis and infection	AXL, TIM-1
		
*Non-enveloped viruses*		
Enterovirus (PV)	Infection	Unknown
Hepatovirus (HAV)	Unknown	TIM-1
Polyomavirus (SV40)	Binding, endocytosis and infection	AXL

Abbreviations: AMAV, amapari virus; CHIKV, chikungunya virus; DENV, dengue virus; EBOV, ebola virus; EEEV, eastern encephalitis equine virus; HAV, hepatitis A virus; LASV, lassa virus; LCMV, lymphocytic choriomeningitis virus; MARV, marburg virus; PV, poliovirus; RRV, Ross river virus; SINV, sindbis virus; SV40, simian virus 40; TCRV, tacaribe virus; VACV MV and EV, vaccinia mature virion and extracellular virion; VSV, vesicular stomatitis virus; WNV, West Nile virus; YFV, yellow fever virus

Listed are the virus families (viruses in parenthesis) experimentally demonstrated to use apoptotic mimicry. The stage of the virus lifecycle facilitated by apoptotic mimicry is listed along with any PS receptors known to be engaged by the various viruses. Refer to text for details and associated references

**Table 1b:** The most deadly infectious agents utilize PS

**Rank 2006**	**Disease**	**Pathogen**	**Class**	**Mortality rate (2006) Deaths ww/ 1000**	**PS involvement**	**Citations**
2	AIDS	HIV	V	49	PS exposure of the virion	Callahan *et al.*^119^
4	Tuberculosis (TB)	*Mycobacterium tuberculosis*	B	27	PS-Decarboxylase	Divangahi *et al.*^120^; Chen *et al.*^121^
5	Malaria	*Plasmodium* spp.	P	22	PS exposure on infected RBC	Eda and Sherman^122^
6	Measles	Paramyxovirus/ Morbillivirus	V	11	Energy consumption —> PS exposure on host cells	Anderton *et al.*^123^
7	Pertussis	*Bordetella pertussis*	B	5	PS-Decarboxylase, PS exposure on host cells	Kawai^124^
8	Tetanus	*Clostridium tetani*	B	4	Tetanustoxin forms ion channels in PS bilayers	Rauch *et al.*^125^
10	Syphilis	Treponema pallidum	B	3	PS exposure	Belisle *et al.*^126^
11	Acute Hepatits B	Hepatitis B Virus	V	2	PS exposure of the virion	De Meyer *et al.*^110^
						
1	Respiratory infections	Multiple pathogens	B, F, V	69	No information due to multiple pathogens	
3	Diarrheal diseases	Multiple pathogens	B, F, V, P	32		
9	Meningitis (all)	Multiple pathogens	B, F, V	3		
12—17	Tropical diseases	Multiple pathogens	B, F, V	2		
						
	Small pox	Variola virus	V	0	PS exposure of the virion	Mercer and Mazzon^127^
	Leishmaniasis	Leishmania spp.	P	0,0069 (2010)	PS exposure of the parasite and of infected neutrophils	van Zandbergen *et al.*^128^
	Trypanosomiasis	*Trypanosoma* spp.	P	0,0014 (2010)	PS exposure of bystander cells	De Souza *et al.*^129^; DaMatta *et al.*^93^
	Ebola	Ebola	V	0,011 (2014)	PS exposure of the virion	Morizone and Chen^65^

Abbreviations: B, bacteria; F, fungi; P, parasite; V, virus

In several of the most deadly infectious diseases, ranked by the death toll worldwide per 1000 people (2006), PS is reportedly involved in the etiopathogenesis. This may reflect the potency of PS bearing microorganisms to evade immune recognition. All statistical data are based on WHO reports from 1993–2014

**Table 2:** Oncology clinical trails assessing Bavituximab

**Indication**	**Phase**	**Trial design**	***n***	**Experimental regimen**	**Results**
*Company sponsored*					
Refractory advanced solid tumors^a^	I	Single-arm, dose escalation	26	Bavituximab monotherapy (0.1, 0.3, 1.0, 3.0 mg/kg)	Well tolerated, pharmacokinetics support weekly dosing
Refractory advanced solid tumors^b^	I	Single-arm	14	Bavituximab+chemotherapy for indication	Well tolerated in combination
Second-line advanced breast cancer^c^	II	Single-arm	46	Bavituximab (3mg/kg)+docetaxel	Overall response rate: 61% Median progression-free survival: 7.4 months (mo.) Median overall survival: 20.7 mo
Front-line non-small cell lung cancer^d^	II	Single-arm	49	Bavituximab (3mg/kg)+carboplatin/paclitaxel	Overall response rate: 41% Median progression-free survival: 6.0 mo Median overall survival: 12.4 mo.
Advanced pancreatic cancer^e^	IIb	Randomized, open label	70	Bavituximab (3mg/kg)+gemcitabine *versus* gemcitabine	Overall response rate: 28.1% *versus* 12.9% Median overall survival: 5.6 *versus*: 5.2 mo
Second-line non-small cell lung cancer^f^	IIb	Randomized, double blind*	121	Bavituximab (3mg/kg or 1mg/kg) or Placebo + docetaxel*	Overall response rate: 17.1% *versus* 11.3%* Median Progression-free survival: 4.2 *versus* 3.9 mo* Median overall survival: 11.7 *versus* 7.3 mo*
					
*Investigator sponsored*					
Front-line HER2-negative breast cancer^g^	I	Single-arm	14	Bavituximab (3mg/kg)+paclitaxel	Overall response rate: 85% Complete response: 15%
Front-line stage IV non-small cell lung cancer^h^	I	Single-arm	25	Bavituximab (3mg/kg)+carboplatin/pemetrexed	Overall response rate: 35% Median progression-free survival: 4.8 mo Median overall survival: 12.2 mo
Front-line hepatocellular carcinoma^i^	I/II	Single-arm, dose escalation	48	Bavituximab (0.1, 0.3, 1.0, 3.0 mg/kg)+sorafenib	Median time to progression 6.7 mo Median overall survival: 6.2 mo

Company-sponsored and investigators-sponsored bavituximab clinical trials. References are indicated where relevant

a Gerber *et al.*^118^

b Digumarti *et al.*,^130^ J. Clin Oncol, 2008, 26 (Suppl) Abstract #3038

c Tabagari *et al.*,^131^ J. Clin Oncol, 2010, 28 (Suppl) Abstract 1042

d Digumarti *et al.*^117^

e Pandya *et al.*,^132^ J. Clin Oncol, 2013, 31 (Suppl) Abstract 4054

f Shtivelband *et al.*,^133^ J. Clin Oncol, 2013, 31 (Suppl) Abstract 8095

g Chalasani *et al.*^116^

h Grilley-Olson *et al.*^134^ Chicago Multidisciplinary Symposium on Thoracic Oncology, 2014 Abstract #215

i Yopp *et al.*,^135^ J. Clin Oncol, 2015, 33 (Suppl) Abstract 4109

* Placebo and 1 mg/kg bavituximab arms were combined for analysis and compared to 3 mg/kg bavituximab arm

## Abbreviations

CTLA4: cytotoxic T-lymphocyte-associated protein 4
Gas6: growth arrest-specific 6
Gla: γ-carboxyglutamic acid
MAMs: mitochondrial- associated membranes
MDL28170: calpain inhibitor
MDSC: myeloid-derived suppressive cells
oxPS: oxidized phosphatidylserine
PS: phosphatidylserine
PC: phosphatidylcholine
PCD: programmed cell death
PD-L1: programmed death-ligand 1
PLSCR: phospholipid scramblase
PTDSS: phosphatidylserine synthase
SNEC: secondary necrotic cells
TAM: Tyro3, Axl, and Mer receptor family
TMEM16: transmembrane protein 16
Xkr8: XK-related Protein 8
Z-VAD-FMK: carbobenzoxy-valyl-alanyl-aspartyl-[O-methyl]-fluoromethylketone, caspase inhibitor
β2GP1: β2-glycoprotein 1
